# Supplementary material for: Combined PIK3CA and SOX2 Gene Amplification Predicts Laryngeal Cancer Risk beyond Histopathological Grading
Source: Int J Mol Sci. 2024 Feb 26;25(5):2695. doi: 10.3390/ijms25052695 (PMC10931972; doi:10.3390/ijms25052695)
Supplement: Supplementary file 1 [file ijms-25-02695-s001.zip › ijms-2828708-supplementary.PDF]

SUPPLEMENTARY INFORMATION

A

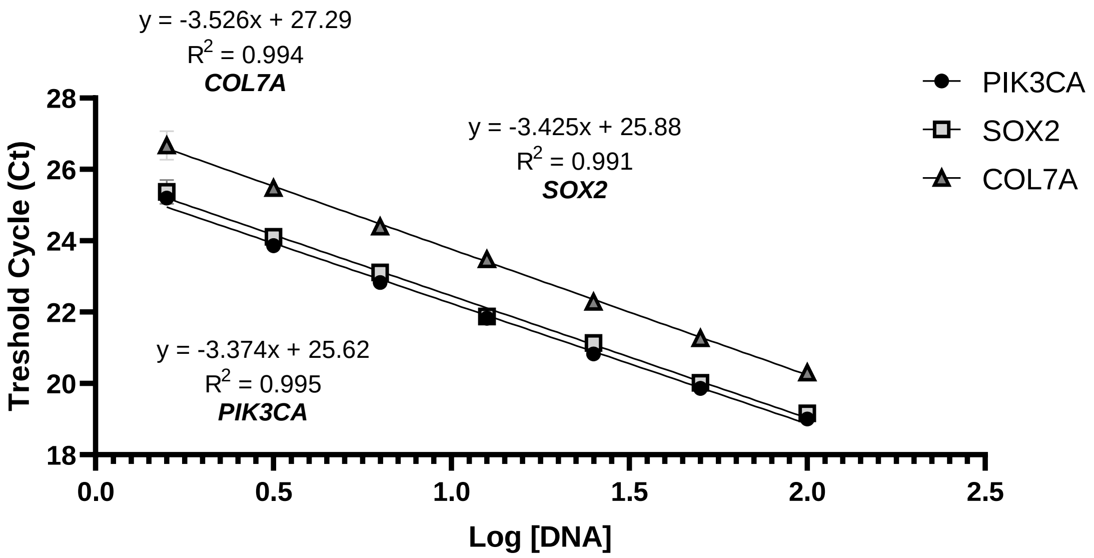

B

Gene	Slope	Efficiency
PIK3CA	-3.374	1.98
SOX2	-3.425	1.96
COL7A	-3.526	1.92

Supplementary Figure S1. Standard curves for the two target genes at 3q26 (*PIK3CA* and *SOX2*) and the control gene *COL7A*. (A) The Log10 DNA amount (in ng per µL) is plotted against threshold cycle (Ct) for different dilutions of genomic DNA in PCR-grade water by real-time PCR. (B) The table shows the efficiency of amplification for each primer pair, calculated using the slope of the corresponding standard curve.

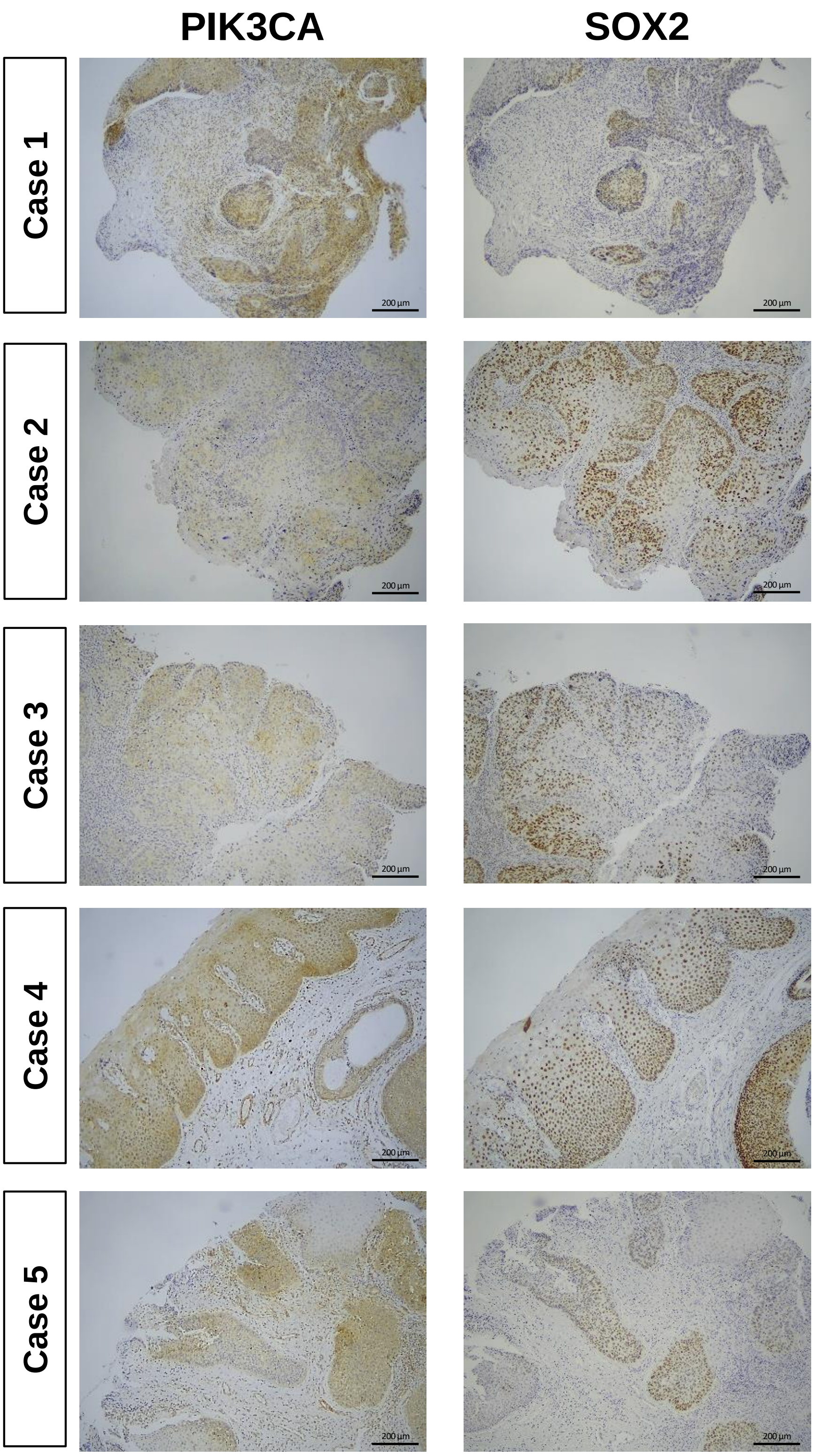

Supplementary Figure S2. Immunohistochemical detection of PIK3CA/p110alpha and SOX2 proteins in a subset of five representative laryngeal dysplasias harboring amplification of *PIK3CA* and *SOX2* genes. Positive cytoplasmic PIK3CA staining and nuclear SOX2 staining were detected in the dysplastic epithelium.

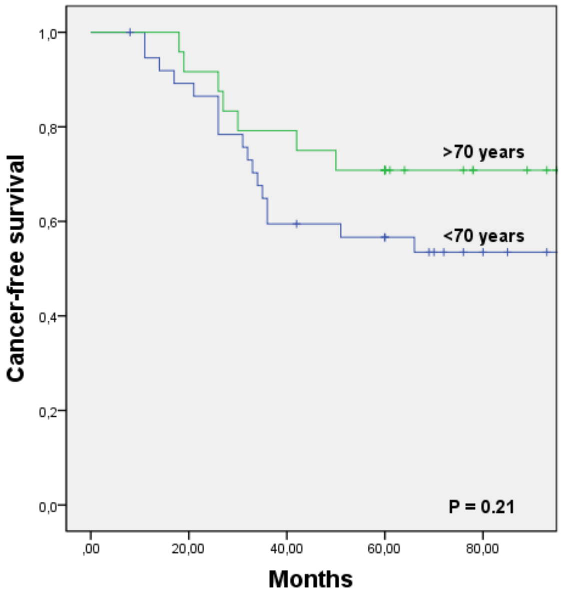

Supplementary Figure S3. Kaplan-Meier cancer-free survival curves in the study series of 62 patients with laryngeal dysplasias categorized by age (<70 years versus >70 years). *p* values were estimated using the log-rank test.

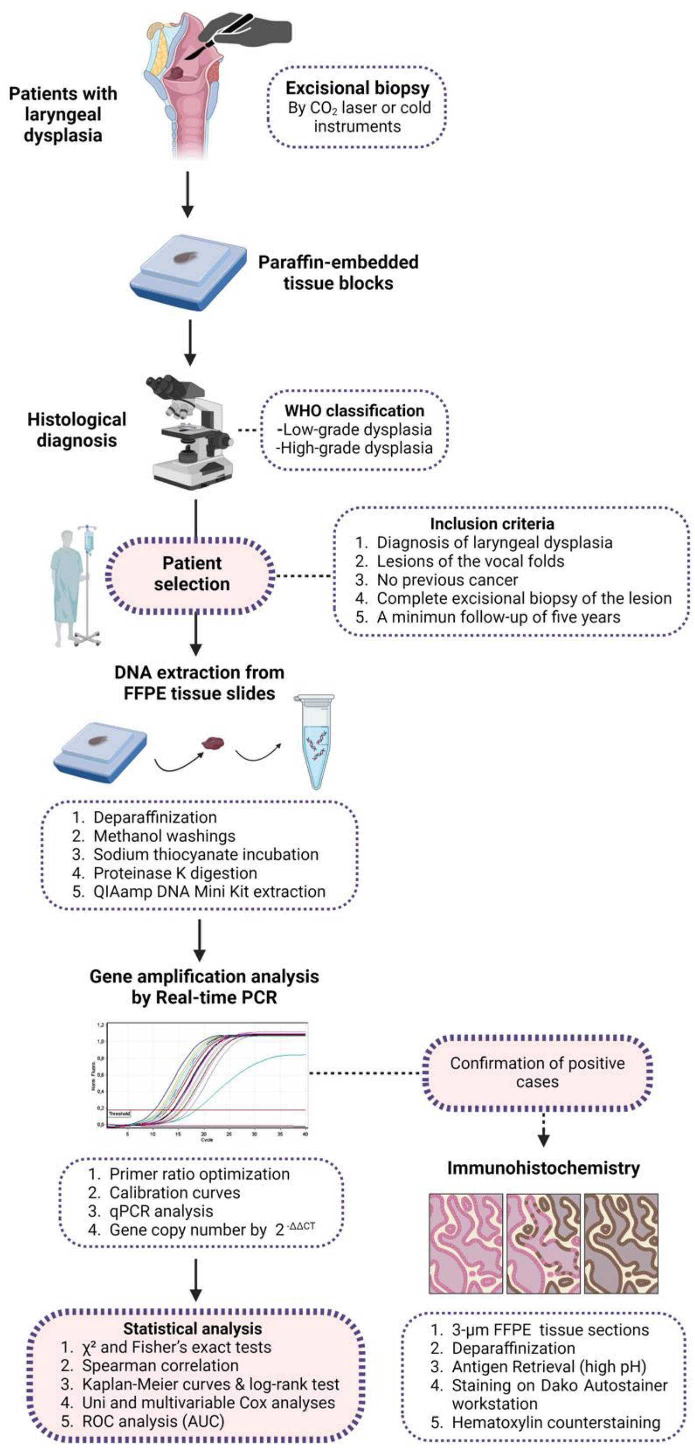

Supplementary Figure S4. Flow chart for the methods used in this study. Figure made in BioRender.com.
